# Total eosinophil count as a biomarker for therapeutic effects of upadacitinib in atopic dermatitis over 48 weeks

**DOI:** 10.3389/fimmu.2024.1365544

**Published:** 2024-04-30

**Authors:** Teppei Hagino, Risa Hamada, Mai Yoshida, Eita Fujimoto, Hidehisa Saeki, Naoko Kanda

**Affiliations:** ^1^ Department of Dermatology, Nippon Medical School Chiba Hokusoh Hospital, Inzai, Japan; ^2^ Department of Dermatology, Nippon Medical School, Tokyo, Japan; ^3^ Fujimoto Dermatology Clinic, Funabashi, Japan

**Keywords:** atopic dermatitis, upadacitinib, Janus kinase inhibitor, biomarker, eosinophil

## Abstract

**Background:**

Atopic dermatitis (AD) is a chronic skin disease characterized by type 2-skewed immune responses, and significantly influenced by cytokines dependent on Janus kinases (JAKs). Upadacitinib, a JAK1 inhibitor, is effective for moderate-to-severe AD. This study aims to identify biomarkers that reflect long-term therapeutic effects of upadacitinib 15 mg or 30 mg.

**Methods:**

A retrospective study from August 2021 to July 2023 included 213 AD patients treated with upadacitinib 15 mg and 70 AD patients with 30 mg. We analyzed eczema area and severity index (EASI), peak pruritus-numerical rating scale (PP-NRS), serum immunoglobulin E (IgE), thymus and activation-regulated chemokine (TARC), lactate dehydrogenase (LDH), and total eosinophil count (TEC) at weeks 0, 4, 12, 24, 36, and 48 of treatment.

**Results:**

Both treatments with upadacitinib 15 mg and 30 mg significantly reduced EASI and PP-NRS scores over week 4 to 48 compared to baseline. Upadacitinib 15 mg or 30 mg treatment significantly decreased TEC compared to baseline through week 4 to 36 or week 4 to 48, respectively. The percent reduction of TEC correlated with those of EASI and PP-NRS through week 4 to 48 of treatment with upadacitinib 15 mg, or through week 12 to 48 with 30 mg, respectively. After adjusting for % reductions of other laboratory markers, the significance of correlations was preserved at weeks 36 and 48 of 15 mg treatment, while at weeks 4 and 36 of 30 mg treatment.

**Conclusion:**

The % reduction of TEC correlated with those of EASI and PP-NRS during upadacitinib treatment, indicating its potential as a biomarker reflecting treatment responses to upadacitinib in AD patients. However, the variability of significant correlation during treatment indicates that further inspection is needed for its usefulness in monitoring responses to upadacitinib treatment for AD.

## Introduction

Atopic dermatitis (AD) is a chronic inflammatory skin disease with type 2-skewed immunity, pruritus, and skin barrier dysfunction (1, 2). Previous studies have shown that the development of AD involves certain cytokines, which intracellularly signal through Janus kinase (JAK)/signal transducer and activator of transcription (STAT) pathways, such as interkeukin (IL)-4, IL-5, IL-13, IL-22, IL-31, or thymic stromal lymphopoietin (TSLP) (3).

Among oral JAK inhibitors approved in Japan, upadacitinib has shown significant therapeutic effectiveness and safety for moderate-to-severe AD, both in clinical trials (4–12) and real-world clinical practice (13). Selective inhibition of JAK1 by upadacitinib targets specific pathways involved in AD, differentiated from first-generation pan-JAK inhibitors such as tofacitinib and ruxolitinib, which inhibit multiple JAK pathways (14).

Previous studies indicate candidate biomarkers reflecting the severity of AD, such as serum immunoglobulin E (IgE), thymus and activation-regulated chemokine (TARC), lactate dehydrogenase (LDH), and total eosinophil count (TEC) (15, 16). On the other hand, the biomarkers reflecting therapeutic effects of upadacitinib have not been established. Monitoring the values of such biomarkers are useful to evaluate the control of AD by upadacitinib. We previously showed that the percentage reduction of TEC correlated with that of eczema area and severity index (EASI) in short-term (≤ 24 weeks) treatment with upadacitinib, indicating that TEC may act as a biomarker reflecting short-term therapeutic effects of upadacitinib (17, 18). However, we have not identified the biomarkers reflecting long-term effectiveness of upadacitinib in AD patients.

This study aims to identify biomarkers that reflect long-term therapeutic effectiveness of upadacitinib 15 mg or 30 mg on clinical signs and pruritus.

## Methods

### Study design and data collection

From August 2021 to September 2023, we administered oral daily upadacitinib 15 mg or 30 mg to 213 or 70 Japanese patients (aged ≥ 12 years) with moderate-to-severe AD, respectively. The diagnosis of AD was made clinically based on the Japanese Atopic Dermatitis Guidelines 2021 (19). These patients had total EASI score ≥ 16 or EASI of head and neck ≥ 2.4. Moderate-to-strongest classes of topical corticosteroids were administered twice daily concomitantly to all the patients. This study was conducted based on the Declaration of Helsinki (2004) and was approved by the Ethics Committee of Nippon Medical School Chiba Hokusoh Hospital. Patients provided written informed consent.

For upadacitinib 15 mg and 30 mg treatment groups, baseline characteristics were recorded and evaluated. These characteristics included sex, age, disease duration of AD, body mass index (BMI), history of bronchial asthma (BA), allergic conjunctivitis, or allergic rhinitis, previous treatment with dupilumab, upadacitinib 15 mg, or baricitinib 4 mg, EASI scores, peak pruritus-numerical rating scale (PP-NRS), investigator’s global assessment (IGA) and values of serum IgE, TARC, LDH, and TEC. We analyzed EASI, IgE, TARC, LDH, and TEC at weeks 0, 4, 12, 24, 36, and 48 of treatment. Patients reported the PP-NRS simultaneously.

In our study, patients who discontinued the treatment were not included in the final analysis. The exclusion of these patients was a deliberate decision to ensure the homogeneity and consistency of the data set, focusing on those who completed the treatment. This approach helps to accurately assess the effects of upadacitinib in patients who adhered to the prescribed treatment regimen.

### Statistical analysis

Results are expressed as mean ± standard deviation for variables with a normal distribution, and as the median and interquartile range for variables with a nonparametric distribution.

Differences in clinical or laboratory indexes at weeks 0, 4, 12, 24, 36, and 48 of treatment were assessed using repeated-measures of analysis of variance for normally distributed variables, and using Friedman’s test for non-parametrically distributed variables. Post-hoc analysis was performed using Bonferroni correction. Differences between two groups were assessed using Student’s *t*-test for variables with a normal distribution, and Mann-Whitney U test for variables with a non-parametric distribution. Statistical significance was set at *p* < 0.05. Correlations between variables were tested using Spearman’s correlation coefficient. We examined if the significant correlation of % reduction of TEC with those of EASI or PP-NRS in univariate analysis might be preserved after adjusting for % reductions of other laboratory markers in multivariate regression analysis.

We further conducted a multiple linear regression analysis to evaluate the independent contributions of % reductions in TEC, TARC, LDH, and IgE to those in EASI or PP-NRS during upadacitinib 15 mg or 30 mg treatment for AD. This analysis included only the variables with significant correlation (p < 0.05) in univariate analysis, and was adjusted for age and sex. Variables with a variance inflation factor >10 were excluded to avoid multicollinearity. We performed all statistical analyses using EZR (Saitama Medical Center, Jichi Medical School).

## Results

### Baseline characteristics of upadacitinib 15 mg and 30 mg treatment groups

Before treatment, BMI, the rates of BA, prior usage of dupilumab, upadacitinib 15 mg, and baricitinib 4 mg, and values of IgE, TARC, and LDH were higher while values of EASI, IGA, and PP-NRS were lower in 30 mg group compared to 15 mg group (**Table 1**).

**Table 1:** Baseline demographic and disease characteristics of patients with atopic dermatitis (*n* = 283).

	Upadacitinib 15 mg (*n* = 213)	Upadacitinib 30 mg (*n* = 70)	*p*
Male sex, *n* (%)	151 (71.0)	53 (75.7)	0.539
Age (years)^a^	36.0 [16.0-51.3]	40.5 [31.5-48.0]	0.346
Body mass index (kg/m^2^) ^a^	22.1 [20.1-25.0]	23.7 [21.5-27.2]	< 0.01**
Disease duration (years) ^a^	28.0 [13.5-44.0]	33.5 [22.0-43.0]	0.0881
Presence of allergic conjunctivitis	31 (46.3)	12 (17.1)	0.571
Presence of allergic rhinitis	67 (31.5)	27 (38.6)	0.307
Presence of bronchial asthma	59 (27.7)	29 (41.4)	0.0374*
Previous dupilumab	12 (5.6)	12 (17.1)	< 0.01**
Previous upadacitinib 15 mg	NA	28 (0.4)	NA
Previous baricitinib 4 mg	4 (1.9)	26 (37.1)	< 0.01**
Clinical indexes			
Total EASI ^a^	23.8 [17.2-32.0]	12.8 [8.6-19.6]	< 0.01**
IGA, *n* (%)			
Mild (score of 2)	19 (8.9)	25 (35.7)	< 0.01^††^
Moderate (score of 3)	115 (54.0)	33 (47.1)	
Severe (score of 4)	79 (37.1)	12 (17.1)	
PP-NRS ^a^	8 [7-9]	6 [3-8]	< 0.01**
Laboratory parameters			
IgE (IU/mL) ^a^	1922.0 [445.8-8369.8]	9592.0 [2124.3-19019.5]	< 0.01**
TARC (pg/mL) ^a^	1299.0 [589.8-3396.3]	5348.5 [1780.0-12812.8]	< 0.01**
LDH (IU/mL) ^a^	227.5 [196.0-287.0]	264.0 [220.0-340.5]	< 0.01**
TEC (/μL) ^a^	450.5 [252.8-687.0]	447.3 [239.2-756.5]	0.514

a Data provided as the median [interquartile range].

* Statistically significant at p < 0.05, ** p < 0.01 by Mann Whitney U test.

^††^ Statistically significant at p < 0.01 by Fisher’s exact test.

EASI, eczema area and severity index; IGA, investigator’s global assessment; PP-NRS, peak pruritus numerical rating scale; IgE, immunoglobulin E; TARC, thymus and activation-regulated chemokine; LDH, lactate dehydrogenase; TEC, total eosinophil count.NA, Not applicable.

### The transition of total EASI and PP-NRS during treatment with upadacitinib 15 mg or 30 mg

**Figure 1:**
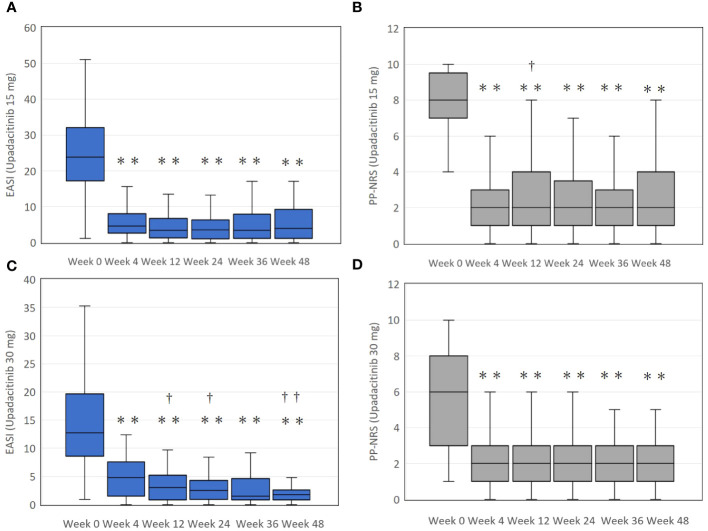
The transition of total eczema area and severity index (EASI) scores **(A, C)**, and the peak pruritus-numerical rating scale (PP-NRS) **(B, D)** during treatment with upadacitinib 15 mg or 30 mg, respectively, in patients with atopic dermatitis. In **(A)** and **(B)**, numbers of patients were 213, 183, 156, 115, 83, or, 63 at week 0, 4, 12, 24, 36, or 48, respectively. In **(C)** and **(D)**, numbers of patients were 70, 66, 59, 49, 39, or 31 at week 0, 4, 12, 24, 36, or 48, respectively. Data are provided as the median [interquartile range]. ** *p* < 0.01 versus values of week 0; †, *p* < 0.05, ††, *p* < 0.01 versus values of week 4, assessed by Friedman’s test with Bonferonni post-hoc test.

In the upadacitinib 15 mg group, EASI significantly decreased at week 4 by median 77.4 interquartile range [70.2-87.3] % of baseline, and plateaued thereafter (**Figure 1A**). PP-NRS significantly decreased at week 4 by 75.0 [57.1-88.9] % of baseline, and plateaued thereafter (**Figure 1B**).

In the upadacitinib 30 mg group, EASI significantly decreased at week 4 by median 68.4 interquartile range [50.0-84.1] % of baseline, and gradually continued to decrease until week 24 with 85.5 [67.3-95.3] % reduction from baseline, and plateaued thereafter (**Figure 1C**). PP-NRS significantly decreased at week 4 by 66.7 [29.8-77.1] % of baseline, and plateaued thereafter (**Figure 1D**).

### The transition of laboratory parameters during treatment with upadacitinib 15 mg or 30 mg

**Figure 2:**
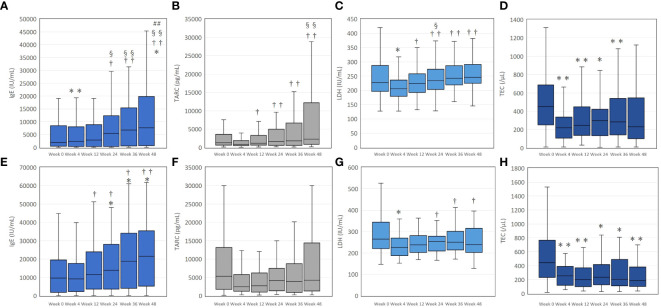
The transition of serum immunoglobulin E (IgE) **(A, E)**, thymus and activation-regulated chemokine (TARC) **(B, F)**, lactate dehydrogenase (LDH) **(C, G)**, and total eosinophil count (TEC) **(D, H)** during treatment with upadacitinib 15 mg or 30 mg, respectively, in patients with atopic dermatitis. In **(A–D)**, numbers of patients were 213, 183, 156, 115, 83, or, 63 at week 0, 4, 12, 24, 36, or 48, respectively. In **(E–H)**, numbers of patients were 70, 66, 59, 49, 39, or 31 at week 0, 4, 12, 24, 36, or 48, respectively. Data are provided as the median [interquartile range]. *, *p* < 0.05, ** *p* < 0.01 versus values of week 0; †, *p* < 0.05, ††, *p* < 0.01 versus values of week 4; §, *p* < 0.05, §§, *p* < 0.01 versus values of week 12; ##, *p* < 0.01 versus values of week 24, assessed by Friedman’s test with Bonferonni post-hoc test.

In the upadacitinib 15 mg group, values of IgE significantly increased at week 4 and 48 compared to baseline (**Figure 2A**). TARC values over weeks 12 to 48 were significantly higher than that of week 4, without significant differences from baseline (**Figure 2B**). Values of LDH significantly decreased at week 4 compared to baseline, without significant differences from baseline at later time-points (**Figure 2C**). TEC significantly decreased at weeks 4, 12, 24, and 36 compared to baseline, without significant difference from baseline at week 48 (**Figure 2D**).

In the upadacitinib 30 mg group, IgE values significantly increased at weeks 24, 36, and 48 compared to baseline (**Figure 2E**). The values of TARC over week 4 to 48 were not significantly different from baseline (**Figure 2F**). LDH significantly decreased at week 4 compared to baseline, without significant differences from baseline at later time-points (**Figure 2G**). TEC significantly decreased over week 4 to 48 compared to baseline (**Figure 2H**).

### Correlation between percent changes of laboratory parameters versus those of EASI during treatment with upadacitinib 15 mg or 30 mg

We then analyzed if percent change of IgE, TARC, LDH, or TEC may correlate with that of EASI. In the upadacitinib 15 mg group (**Table 2**), the percent change of IgE positively correlated with that of EASI at week 12 and 24. The percent change of TARC positively correlated with that of EASI at week 12, 24, 36 and 48. The percent reduction of LDH positively correlated with that of EASI at week 12 and 24. The percent reduction of TEC positively correlated with that of EASI at week 4, 12, 24, 36 and 48.

**Table 2:** Correlations between percent reductions of laboratory parameters versus those of EASI at weeks 4, 12, 24, 36, or 48 of treatment with upadacitinib 15 mg in patients with atopic dermatitis (*n* = 213 at week 0).

Laboratory Parameters	Week 4 (*n* = 183)		Week 12 (*n* = 156)		Week 24 (*n* = 115)		Week 36 (*n* =83)		Week 48 (*n* = 63)	
	Rho	*p*	Rho	*p*	Rho	*p*	Rho	*p*	Rho	*p*
IgE (IU/mL) ^a^	0.079	0.345	0.339	< 0.01**	0.261	< 0.01**	0.22	0.113	0.247	0.0843
TARC (pg/mL) ^a^	0.109	0.185	0.45	< 0.01**	0.41	< 0.01**	0.406	< 0.01**	0.579	< 0.01**
LDH (IU/mL) ^a^	0.0687	0.376	0.293	< 0.01**	0.232	0.0172*	0.0602	0.636	0.218	0.118
Total eosinophil count (/μL) ^a^	0.275	< 0.01**	0.429	< 0.01**	0.377	< 0.01**	0.421	< 0.01**	0.518	< 0.01**

Correlations between variables were examined using Spearman’s correlation coefficient.

* Statistically significant at p < 0.05, ** at p < 0.01.

EASI, eczema area and severity index; IgE, immunoglobulin E; TARC, thymus and activation-regulated chemokine; LDH, lactate dehydrogenase.

In the upadacitinib 30 mg group (**Table 3**), the percent change of IgE positively correlated with that of EASI at week 24. The percent reduction of TARC positively correlated with that of EASI at week 12, 24, and 36. The percent reduction of LDH positively correlated with that of EASI at week 24. The percent reduction of TEC positively correlated with that of EASI at week 12, 24, 36 and 48.

**Table 3:** Correlations between percent reductions of laboratory parameters versus those of EASI at weeks 4, 12, 24, 36, or 48 of treatment with upadacitinib 30 mg in patients with atopic dermatitis (*n* = 70 at week 0).

Laboratory Parameters	Week 4 (*n* = 66)		Week 12 (*n* = 59)		Week 24 (*n* = 49)		Week 36 (*n* = 39)		Week 48 (*n* = 31)	
	Rho	*p*	Rho	*p*	Rho	*p*	Rho	*p*	Rho	*p*
IgE (IU/mL) ^a^	0.0758	0.575	0.231	0.0998	0.483	< 0.01**	0.23	0.191	0.249	0.253
TARC (pg/mL) ^a^	0.102	0.446	0.284	0.0412*	0.515	< 0.01**	0.341	0.0486*	0.34	0.104
LDH (IU/mL) ^a^	0.155	0.241	0.188	0.157	0.316	0.0346*	0.0352	0.841	0.193	0.336
Total eosinophil count (/μL) ^a^	0.251	0.0573	0.382	< 0.01**	0.391	< 0.01**	0.432	< 0.01**	0.525	< 0.01**

Correlations between variables were examined using Spearman’s correlation coefficient.

* Statistically significant at p < 0.05, ** at p < 0.01.

EASI, eczema area and severity index; IgE, immunoglobulin E; TARC, thymus and activation-regulated chemokine; LDH, lactate dehydrogenase.

### Correlation between percent changes of laboratory parameters versus those of PP-NRS during treatment with upadacitinib 15 mg or 30 mg

We then analyzed if percent change of IgE, TARC, LDH, or TEC may correlate with that of PP-NRS. In the upadacitinib 15 mg group (**Table 4**), the percent change of IgE positively correlated with that of PP-NRS at week 12, 24, 36 and 48. The percent change of TARC positively correlated with that of PP-NRS at weeks 4, 12, 24, 36 and 48. The percent reduction of LDH positively correlated with that of PP-NRS at week 12. The percent reduction of TEC positively correlated with that of PP-NRS at week 4, 12, 24, 36 and 48.

**Table 4:** Correlations between percent reductions of laboratory parameters versus those of PP-NRS at weeks 4, 12, 24, 36, or 48 of treatment with upadacitinib 15 mg in patients with atopic dermatitis (*n* = 213 at week 0).

Laboratory Parameters	Week 4 (*n* = 183)		Week 12 (*n* = 156)		Week 24 (*n* = 115)		Week 36 (*n* = 83)		Week 48 (*n* = 63)	
	Rho	*p*	Rho	*p*	Rho	*p*	Rho	*p*	Rho	*p*
IgE (IU/mL) ^a^	0.0734	0.38	0.428	< 0.01**	0.3	< 0.01**	0.382	< 0.01**	0.398	< 0.01**
TARC (pg/mL) ^a^	0.31	< 0.01**	0.467	< 0.01**	0.406	< 0.01**	0.555	< 0.01**	0.466	< 0.01**
LDH (IU/mL) ^a^	0.0669	0.389	0.253	< 0.01**	0.13	0.192	0.214	0.0888	0.211	0.129
Total eosinophil count (/μL) ^a^	0.314	< 0.01**	0.257	< 0.01**	0.398	< 0.01**	0.527	< 0.01**	0.517	< 0.01**

Correlations between variables were examined using Spearman’s correlation coefficient.

* Statistically significant at p < 0.05, ** at p < 0.01.

PP-NRS, peak pruritus-numerical rating scale; IgE, immunoglobulin E; TARC, thymus and activation-regulated chemokine; LDH, lactate dehydrogenase.

In the upadacitinib 30 mg group (**Table 5**), the percent change of IgE positively correlated with that of PP-NRS at week 36. The percent reduction of TARC positively correlated with that of PP-NRS at week 4, 12, 24 and 36. The percent reduction of LDH positively correlated with that of PP-NRS at week 4 and 48. The percent reduction of TEC positively correlated with that of EASI at week 12, 24, 36 and 48.

**Table 5:** Correlations between percent reductions of laboratory parameters versus those of PP-NRS at weeks 4, 12, 24, 36, or 48 of treatment with upadacitinib 30 mg in patients with atopic dermatitis (*n* = 70 at week 0).

Laboratory Parameters	Week 4 (*n* = 66)		Week 12 (*n* = 59)		Week 24 (*n* = 49)		Week 36 (*n* = 39)		Week 48 (*n* = 31)	
	Rho	*p*	Rho	*p*	Rho	*p*	Rho	*p*	Rho	*p*
IgE (IU/mL) ^a^	0.0472	0.73	0.143	0.313	0.279	0.0736	0.414	0.0133*	0.262	0.293
TARC (pg/mL) ^a^	0.346	< 0.01**	0.355	< 0.01**	0.456	< 0.01**	0.445	< 0.01**	0.304	0.158
LDH (IU/mL) ^a^	0.27	0.0404*	0.223	0.095	0.247	0.102	0.107	0.535	0.449	0.0212*
Total eosinophil count (/μL) ^a^	0.165	0.221	0.484	< 0.01**	0.396	< 0.01**	0.515	< 0.01**	0.65	< 0.01**

Correlations between variables were examined using Spearman’s correlation coefficient.

* Statistically significant at p < 0.05, ** at p < 0.01.

PP-NRS, peak pruritus-numerical rating scale; IgE, immunoglobulin E; TARC, thymus and activation-regulated chemokine; LDH, lactate dehydrogenase.

Although % change of IgE positively correlated with that of PP-NRS (**Table 4**) at week 48 of upadacitinib 15 mg treatment, serum IgE increased (**Figure 2A**) while PP-NRS reduced (**Figure 1B**) compared to baseline. The discrepant results are explained as follows: when % reduction of IgE (x-axis) and that of PP-NRS (y-axis) for each patient is plotted at week 48 of 15 mg upadacitinib treatment (**Supplementary Figure 1A**), % reduction of PP-NRS was median 75.0 (ranging from -80 to 100)%, mostly > 0 while that of IgE was median -81.9 (ranging from -2480.8 to 81) %, mostly < 0, ranging from minus to plus numbers. In this figure, increasing % change of IgE (x-axis) from minus to plus numbers across 0-point was associated with increasing % reduction of PP-NRS (y-axis), mostly > 0, resulting in significant positive correlation between the two variables. Similar trend was revealed in % changes of TARC and EASI at week 48 of upadacitinib 15 mg treatment (**Supplementary Figure 1B**). As shown in these figures, at later time-points (weeks 24, 36, or 48) of upadacitinib 15 or 30 mg treatment, serum IgE or TARC either increased (% reduction < 0) or decreased (% reduction > 0) even in the patients whose EASI or PP-NRS decreased (% reduction > 0) compared to baseline. These results indicate that the transitions of serum IgE and TARC may not reflect those of EASI and PP-NRS at later time-points (weeks 24, 36, or 48) of upadacitinib treatment.

On the other hand, TEC reduced almost all over weeks 4 to 48 of 15 mg and 30 mg upadacitinib treatment compared to baseline (**Figures 2D, H**) in parallel with EASI (**Figures 1A, C**) or PP-NRS (**Figure 1B, D**). Further, the % reduction of TEC positively correlated with those of EASI and PP-NRS almost all over 48 weeks of treatment with 15 mg and 30 mg upadacitinib (**Tables 2**–**5**). The results totally indicate that TEC may act as an indicator of therapeutic effects of upadacitinib on clinical signs and pruritus in AD.

### Multivariate analysis for the correlation of %reduction in TEC with that in EASI or PP-NRS adjusted for those of other laboratory markers

We further examined if the significant correlation of %reduction of TEC with that of EASI or PP-NRS by upadacitinib treatment might be preserved after adjusting for those of other laboratory markers (**Table 6**).

**Table 6:** Correlation of % reduction of TEC with % reduction of EASI or PP-NRS adjusted for % reductions of IgE, LDH, and TARC.

			Correlation with % reduction of EASI		Correlation with % reduction of PP-NRS	
Upadacitinib dose	Week	n	Adjusted R	*p*	Adjusted R	*p*
15 mg	Week 4	183	0.1253	0.6962	0.1206	0.7447
	Week 12	156	0.0903	0.9232	0.1218	0.6752
	Week 24	115	0.2162	0.08232	0.2089	0.09597
	Week36	83	0.784	< 0.01**	0.6416	< 0.01**
	Week48	63	0.8417	< 0.01**	0.6639	< 0.01**
30 mg	Week 4	66	0.2665	< 0.01**	0.5375	< 0.01**
	Week 12	59	0.04969	0.1735	0.3187	0.05965
	Week 24	49	0.3036	0.1087	0.2966	0.1164
	Week36	39	0.5487	< 0.01**	0.5719	< 0.01**
	Week48	31	0.1927	0.5266	0.4444	0.9001

** Statistically significant at p < 0.01

EASI, eczema area and severity index; PP-NRS, peak pruritus numerical rating scale; IgE, immunoglobulin E; TARC, thymus and activation-regulated chemokine; LDH, lactate dehydrogenase; TEC, total eosinophil count.

In 15 mg treatment group, the significant correlations were preserved at week 36 and 48, later stages of the treatment while in 30 mg group, those were preserved at week 4 and 36, early- and mid-treatment stages. At the other time-points, the significance of correlation was lost.

### Multiple linear regression analysis to assess the independent contributions of % reductions of laboratory markers to the % reduction of EASI or PP-NRS

A comprehensive multiple linear regression analysis was conducted to ascertain the independent contributions of % reductions in laboratory indexes (TEC, TARC, LDH, and IgE) to % reduction in EASI or PP-NRS by treatment with upadacitinib 15 mg or 30 mg. This analysis, adjusted for age and sex as confounding factors, identified independent contribution at several time-points of treatment. In upadacitinib 15 mg treatment, the independent contribution to % reduction of EASI (**Supplementary Table 1**) was revealed in % reduction of TARC at week 24, 36, and 48, and in % reduction of LDH at week 12 and 24. In upadacitinib 30 mg treatment, the independent contribution to % reduction of EASI was revealed in % reduction of TARC at week 12 and 36.

In upadacitinib 15 mg treatment, the independent contribution to % reduction of PP-NRS (**Supplementary Table 2**) was revealed in % reduction of TARC at week 4, 24, and 48, and in % reduction of LDH at week 4. In upadacitinib 30 mg treatment, the independent contribution to % reduction of EASI was revealed in % reduction of LDH at week 4. Independent contribution to % reduction of EASI or PP-NRS was not revealed in % reduction of TEC or IgE.

## Discussion

The percent reduction of TEC positively correlated with those of EASI and PP-NRS through week 4 to 48 of treatment with upadacitinib 15 mg, or through week 12 to 48 with 30 mg, respectively.

These findings align with our previous results, showing a decrease in TEC or eosinophil/lymphocyte ratio in parallel with EASI at week 4, 12, and 24 of upadacitinib 15 mg treatment (17, 18). The correlation of the transition of TEC with those of EASI and PP-NRS in upadacitinib treatment suggest that eosinophils may contribute to rash and pruritus in AD, and can be the target of upadacitinib treatment. Eosinophils produce IL-13 (20) and IL-31 (21), promoting type 2 inflammation, and secrete chemokines CCL18, CXCL1, or CCL2 promoting the recruitment of CLA+ memory T cells, basophils, or dendritic cells, respectively (22). At the upper dermis of AD skin lesions, eosinophils and neurons colocalize (23), and reciprocally interact via releasing mediators, which might potentiate their growth or survival and pruritus. Eosinophils secrete brain-derived growth factor (23) and IL-31 (21), which promote the proliferation of sensory neurons, inducing hyperinnervation. Interleukin-31 also binds to its receptor on sensory nerve endings, and induces itch sensation (24). Eosinophils secrete major basic protein, which potentiates the survival of nerve cells (25), and induces their release of substance P (26). Substance P in turn facilitates chemotaxis and survival of eosinophils (27).

The decrease in TEC by upadacitinib treatment may involve IL-5. Interleukin-5 critically regulates expression of genes involved in proliferation, survival, maturation, and effector functions of eosinophils (28). Interleukin-5 receptor (R) consists of IL-5Rα and βC. Both JAK1 and JAK2 are required for IL-5-induced tyrosine phosphorylation of βC and signal transduction in eosinophils (29). Upadacitinib might suppress the IL-5-induced phosphorylation and activation of JAK1 and/or JAK2 in eosinophils, and resultantly suppress the gene expression involved in proliferation and survival, leading to the decrease in TEC. Interleukin-31 also signals through JAK1 in eosinophils, and suppresses their apoptosis, contributing to their survival (22). Thus the inhibition on the anti-apoptotic effects of IL-31 may also be involved in upadacitinib-mediated decrease in TEC.

In the present study, the significance of correlations of TEC with EASI or PP-NRS was preserved after adjusting for other laboratory indexes, at week 36 and 48 of 15 mg upadacitinib treatment, and at week 4 and 36 of 30 mg treatment. However, at other time-points, the significance was lost (**Table 6**). Further, % reduction of TEC did not independently contribute to % reductions of EASI or PP-NRS in upadacitinib treatment in a linear multivariable regression analyses (**Supplementary Tables 1**, **2**). These results indicate that correlation of TEC with clinical improvement was not constant, and may be influenced by the heterogeneous responsiveness of patients’ eosinophils to upadacitinib. At time-points with preserved significant correlation, a larger proportion of patients might have high susceptibility to the effects of upadacitinib to reduce survival of eosinophils. However, at time-points with insignificant correlation, the proportion of patients with high susceptibility may be smaller. The ability of TEC to reflect clinical improvement by upadacitinib may be variable. Therefore, while TEC might be a potential biomarker to reflect treatment responses to upadacitinib, its utility is not uniformly applicable across all treatment periods. A comprehensive approach with multiple biomarkers may be required to monitor the clinical improvement by upadacitinib treatment for AD.

We previously studied the transition of TEC during systemic treatments for AD, other than upadacitinib. During the treatment with another JAK1 inhibitor abrocitinib, TEC tended to decrease with clinical improvement in AD patients (30). TEC was significantly reduced at week 4 of treatment with JAK1/2 inhibitor baricitinib, compared to baseline (31). These results indicate that treatment with JAK inhibitors might commonly reduce TEC in AD patients. In contrast, several studies reported that TEC transiently increased during treatment with IL-4Rα antibody dupilumab for AD patients (32, 33). It is hypothesized that dupilumab might inhibit IL-4/13-induced expression of eotaxin-3 in fibroblasts or of VCAM-1 in endothelial cells (34, 35), which might prevent transmigration of eosinophils across the vascular bed into tissues such as skin or airway, and result in their accumulation in the bloodstream. In asthma model mice, deficiency of both IL-4 and IL-13 markedly reduced the number of eosinophils in airway, and induced blood eosinophilia (36, 37). Similarly, treatment with anti-IL-13 antibodies tralokinumab and lebrikizumab transiently increased TEC (38, 39). These results indicate that treatments with JAK inhibitors might decrease TEC while treatments targeting IL-4 and/or IL-13 might increase TEC in AD patients. These variable responses in TEC according to the different treatments for AD suggest the need for further increased collection of real-world data and precise investigation of the mechanisms.

Serum IgE and TARC increased after long-term treatment with upadacitinib (week 24, 36 or 48) in this study. Several mechanisms can be hypothesized for the results. Firstly, the increase in TARC may involve IL-17A which suppresses TARC production in dendritic cells (40). It is reported that siRNA-mediated silencing of *JAK1* reduced *in vitro* IL-17A production in CD3/28-stimulated CD4+ human T cells in the presence of abundant IL-2, IL-1β, IL-6, and IL-23 (41). A recent study also reported that serum IL-17A levels decreased after upadacitinib treatment in patients with AD (42). Thus the possible decrease in endogenous IL-17A levels by upadacitinib via inhibition of JAK1 might attenuate the suppressive effects of IL-17A on TARC production, leading to the increase of serum TARC levels after long-term treatment with upadacitinib.

Secondly, the increase in serum IgE may involve IL-21 which suppresses IgE class switching in B cells (43). Interleukin-21 binds its receptor, IL-21R/γC and signals through JAK1/JAK3 (44). Upadacitinib inhibits IL-21-induced phosphorylation of STAT3 dependent on JAK1/3 in CD4+ T cells or natural killer cells (45). *IL21R* knockout mice exhibit increased IgE responses after immunization (46). Taken together, oral upadacitinib treatment may block the IL-21-mediated suppression of IgE class switching via inhibition of JAK1, leading to the increase in serum IgE levels.

Serum TARC increased though EASI and PP-NRS reduced at later phases of upadacitinib 15 mg treatment (weeks 24, 36, or 48) (**Figure 2**). However, % reduction of TARC significantly correlated with those of EASI or PP-NRS in both univariate (**Tables 2**-**5**) and multivariate analyses (**Supplementary Tables 1**, **2**) at several time-points of later phases of upadacitinib 15 mg treatment. This discrepancy indicates that the transition of serum TARC may not reflect those of EASI and PP-NRS at least at later phases of upadacitinib treatment.

This study has several limitations. Firstly, the participants of this study were limited to Japanese, and further studies should be performed on the patients with different races. Secondly, our study measured circulating eosinophil levels in the blood; however, IL-5 primarily recruits eosinophils to inflamed AD skin. Future studies must measure eosinophil counts in the skin tissue samples from patients treated with upadacitinib to fully understand the impact of the drug on eosinophil migration and activity in affected tissues. Thirdly, the number of patients treated with upadacitinib 30 mg was much smaller compared to that with 15 mg.

In conclusion, the % reduction of TEC correlated with those of EASI and PP-NRS during upadacitinib treatment for AD, indicating that TEC might act as a biomarker reflecting treatment responses to upadacitinib in AD patients. However, the variability of significant correlation during treatment indicates that further inspection is needed for its usefulness in monitoring responses to upadacitinib treatment for AD.

## Data availability statement

The raw data supporting the conclusions of this article will be made available by the authors, without undue reservation.

## Ethics statement

This study was conducted in accordance with the Declaration of Helsinki, and approved by the Ethics Committee of Nippon Medical School Chiba Hokusoh Hospital (protocol code H-2022-945, approved on 10 February, 2022). The studies were conducted in accordance with the local legislation and institutional requirements. Written informed consent for participation in this study was provided by the participants’ legal guardians/next of kin.

## Author contributions

TH: Conceptualization, Writing – original draft, Writing – review & editing. RH: Formal analysis, Writing – review & editing. MY: Formal analysis, Writing – review & editing. EF: Writing – review & editing. HS: Writing – review & editing. NK: Writing – review & editing.
