# Supplementary material for: Total eosinophil count as a biomarker for therapeutic effects of upadacitinib in atopic dermatitis over 48 weeks
Source: Front Immunol. 2024 Apr 30;15:1365544. doi: 10.3389/fimmu.2024.1365544 (PMC11091278; doi:10.3389/fimmu.2024.1365544)
Supplement: Supplementary file 1 [file DataSheet_1.docx]

Supplementary Material

Total eosinophil count as a biomarker for therapeutic effects of upadacitinib in atopic dermatitis over 48 weeks

Teppei Hagino^1*^, Risa Hamada^2^, Mai Yoshida^2^, Eita Fujimoto^3^, Hidehisa Saeki^2^, Naoko Kanda^1^

^1^Department of Dermatology, Nippon Medical School Chiba Hokusoh Hospital, Inzai, Japan

^2^Department of Dermatology, Nippon Medical School, Tokyo, Japan

^3^Fujimoto Dermatology Clinic, Funabashi, Japan

*** Correspondence:**Teppei Hagino, MD, Department of Dermatology, Nippon Medical School Chiba Hokusoh Hospital, Kamagari 1715, Inzai, Chiba 270-1694, Japan

Tel: +81 476 99 1111; Fax: +81 476 99 1911; E-mail: teppei-hagino@nms.ac.jp


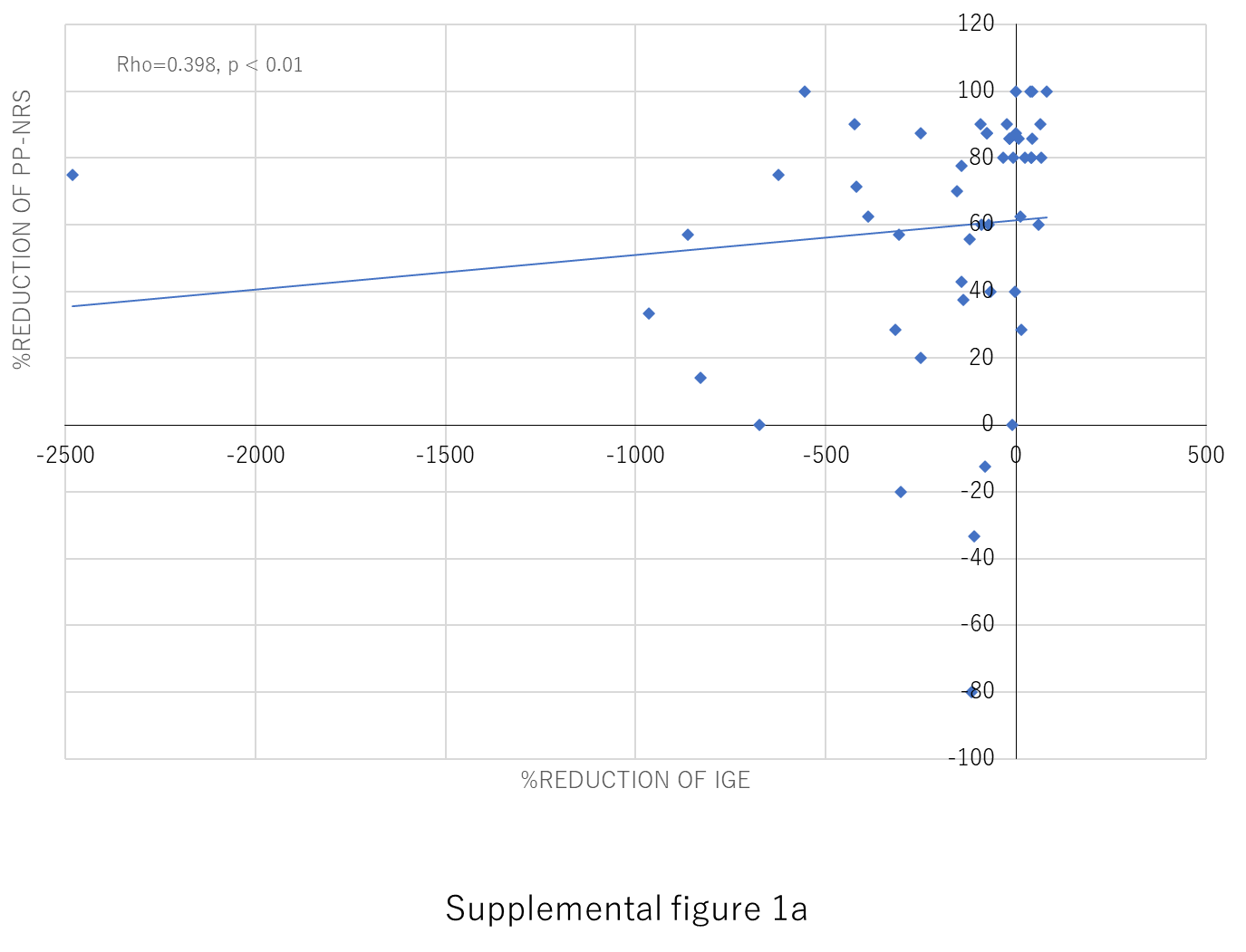

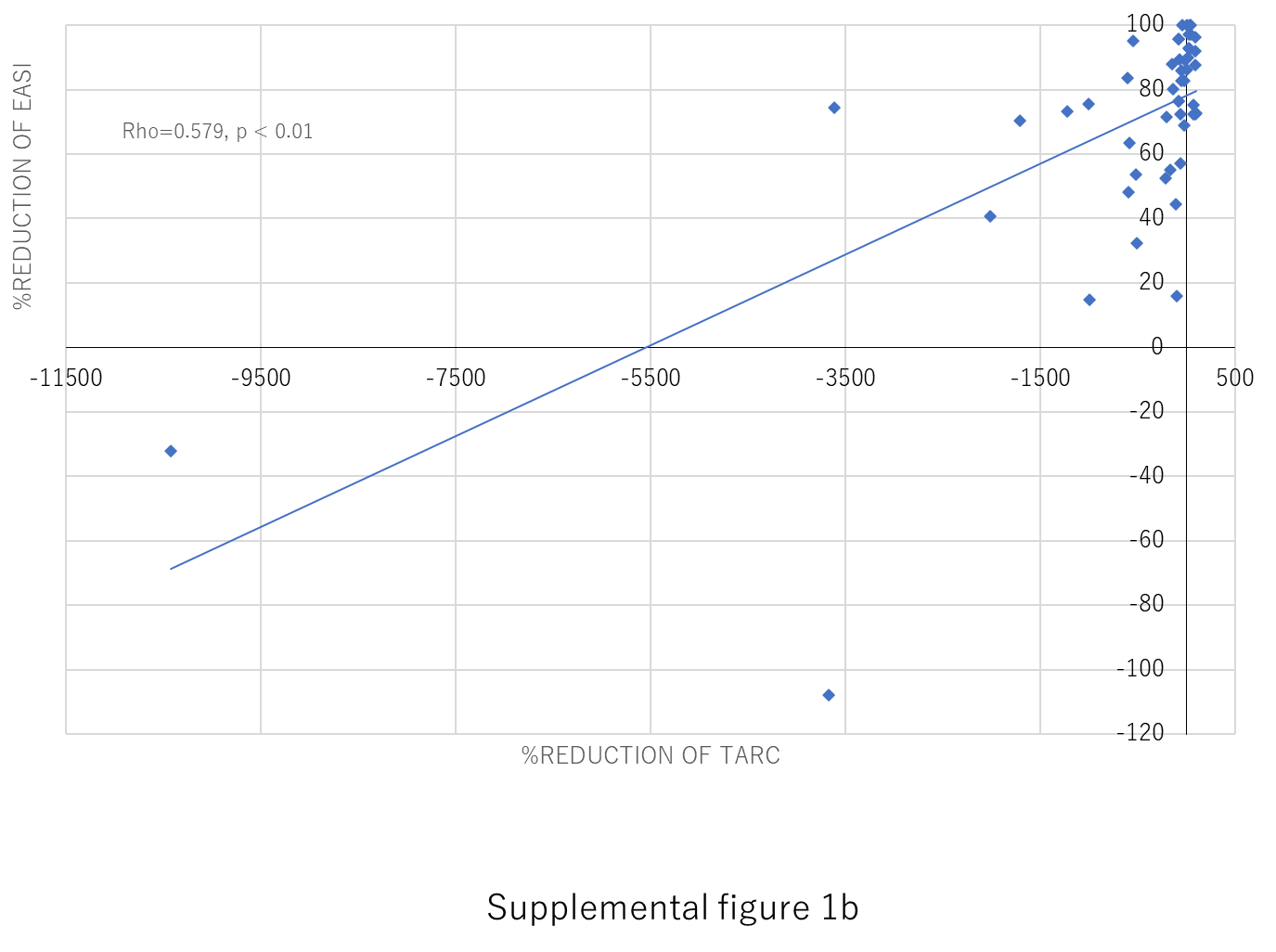


**Supplementary Figure 1.** This figure presents the correlations between percent reductions of serum immunoglobulin E (IgE) and peak pruritus-numerical rating scale (PP-NRS) (Figure 1a), or between those of thymus and activation-regulated chemokine (TARC) and eczema area and severity index (EASI) (Figure 1b), at week 48 of upadacitinib 15 mg treatment.
