# Supplementary material for: Total eosinophil count as a biomarker for therapeutic effects of upadacitinib in atopic dermatitis over 48 weeks
Source: Front Immunol. 2024 Apr 30;15:1365544. doi: 10.3389/fimmu.2024.1365544 (PMC11091278; doi:10.3389/fimmu.2024.1365544)
Supplement: Supplementary file 2 [file Table_1.docx]

| Supplemental Table 1: Multiple linear regression analysis to assess the independent contributions of % reductions of laboratory indexes to the % reduction of EASI | | | | | | | | | | | | | | | | | | | | | |
| --- | --- | --- | --- | --- | --- | --- | --- | --- | --- | --- | --- | --- | --- | --- | --- | --- | --- | --- | --- | --- | --- |
|  |  | Percent reduction of EASI at week 4 | | | | Percent reduction of EASI at week 12 | | | | Percent reduction of EASI at week 24 | | | | Percent reduction of EASI at week 36 | | | | Percent reduction of EASI at week 48 | | | |
| Upadacitinib dose |  | β coefficient | Standard error | *t* | *p* | β coefficient | Standard error | *t* | *p* | β coefficient | Standard error | *t* | *p* | β coefficient | Standard error | *t* | *p* | β coefficient | Standard error | *t* | *p* |
| 15 mg | (Intercept) | 76 | 1.26 | 60.1 | < 0.01 | 77.1 | 2.08 | 36.9 | < 0.01 | 82.7 | 2.38 | 34.7 | < 0.01 | 74.4 | 3.69 | 20.1 | < 0.01 | 74.9 | 3.72 | 20.1 | < 0.01 |
|  | % reduction of IgE | NA | | | | 0.0108 | 0.0157 | 0.687 | 0.493 | -0.0109 | 0.0074 | -1.48 | 0.141 | NA | | | | | | | |
|  | % reduction of TARC |  |  |  |  | 0.0101 | 0.008157 | 1.24 | 0.217 | 0.0249 | 0.00468 | 5.31 | < 0.01** | 0.0253 | 0.00489 | 5.18 | < 0.01** | 0.0103 | 0.00221 | 4.67 | < 0.01** |
|  | % reduction of LDH |  |  |  |  | 0.243 | 0.0853 | 2.85 | < 0.01** | 0.24 | 0.101 | 2.36 | 0.0203* | NA | | | | | | | |
|  | % reduction of TEC | 0.017 | 0.0139 | 1.22 | 0.223 | 0.00272 | 0.003897 | 0.698 | 0.486 | -0.00163 | 0.00791 | -0.206 | 0.837 | 0.284 | 0.0536 | 5.3 | < 0.01** | 0.171 | 0.0353 | 4.84 | < 0.01** |
| 30 mg | (Intercept) | NA | | | | 72.7 | 3.51 | 20.6 | < 0.01 | 80.3 | 6.02 | 13.3 | < 0.01 | 86.7 | 8.49 | 10.2 | < 0.01 | 64.5 | 19.1 | 3.36 | < 0.01 |
|  | % reduction of IgE |  |  |  |  | NA | | | | 0.065 | 0.0401 | 1.62 | 0.113 | NA | | | | | | | |
|  | % reduction of TARC |  |  |  |  | 0.138 | 0.037 | 3.73 | < 0.01** | 0.00435 | 0.0383 | 0.113 | 0.91 | 0.261 | 0.0365 | 7.16 | < 0.01** | NA | | | |
|  | % reduction of LDH |  |  |  |  | NA | | | | 0.295 | 0.229 | 1.28 | 0.206 | NA | | | | | | | |
|  | % reduction of TEC |  |  |  |  | 0.056 | 0.0456 | 1.22 | 0.225 | 0.0391 | 0.0686 | 0.571 | 0.571 | -0.0157 | 0.1 | -0.156 | 0.876 | 0.0246 | 0.156 | 0.157 | 0.87 |
| * Statistically significant at *p* < 0.05, ** at *p* < 0.01.  EASI, eczema area and severity index; PP-NRS, peak pruritus numerical rating scale; IgE, immunoglobulin E; TARC, thymus and activation-regulated chemokine; LDH, lactate dehydrogenase; TEC, total eosinophil count; NA, not applicable. | | | | | | | | | | | | | | | | | | | | | |
