# Supplementary material for: Total eosinophil count as a biomarker for therapeutic effects of upadacitinib in atopic dermatitis over 48 weeks
Source: Front Immunol. 2024 Apr 30;15:1365544. doi: 10.3389/fimmu.2024.1365544 (PMC11091278; doi:10.3389/fimmu.2024.1365544)
Supplement: Supplementary file 3 [file Table_2.docx]

| Supplemental Table 2: Multiple linear regression analysis to assess the independent contributions of % reductions of laboratory indexes to the % reduction of PP-NRS | | | | | | | | | | | | | | | | | | | | | |
| --- | --- | --- | --- | --- | --- | --- | --- | --- | --- | --- | --- | --- | --- | --- | --- | --- | --- | --- | --- | --- | --- |
|  |  | Percent reduction of PP-NRS at week 4 | | | | Percent reduction of PP-NRS at week 12 | | | | Percent reduction of PP-NRS at week 24 | | | | Percent reduction of PP-NRS at week 36 | | | | Percent reduction of PP-NRS at week 48 | | | |
| Upadacitinib dose |  | β coefficient | Standard error | *t* | *p* | β coefficient | Standard error | *t* | *p* | β coefficient | Standard error | *t* | *p* | β coefficient | Standard error | *t* | *p* | β coefficient | Standard error | *t* | *p* |
| 15 mg | (Intercept) | 69.7 | 2.05 | 33.8 | < 0.01 | 57 | 5.33 | 10.6 | < 0.01 | 65.2 | 3.91 | 16.6 | < 0.01 | 63.2 | 5.83 | 10.8 | < 0.01 | 62.4 | 6.49 | 9.62 | < 0.01 |
|  | % reduction of IgE | NA | | | | 0.0474 | 0.0402 | 1.17 | 0.241 | 0.00401 | 0.0123 | 0.324 | 0.746 | 0.00041 | 0.019 | 0.0215 | 0.982 | 0.00491 | 0.0137 | 0.357 | 0.722 |
|  | % reduction of TARC | 0.113 | 0.0309 | 3.66 | < 0.01** | 0.0074 | 0.0208 | 0.355 | 0.723 | 0.0212 | 0.00618 | 3.43 | < 0.01** | 0.0174 | 0.00858 | 2.03 | 0.0476* | 0.00685 | 0.00363 | 1.88 | 0.0659 |
|  | % reduction of LDH | NA | | | | 0.383 | 0.218 | 1.75 | 0.0814 | NA | | | | | | | | | | | |
|  | % reduction of TEC | 0.0162 | 0.0212 | 0.767 | 0.443 | 0.0137 | 0.00995 | 1.38 | 0.169 | 0.00372 | 0.013 | 0.286 | 0.775 | 0.126 | 0.0866 | 1.45 | 0.151 | 0.0366 | 0.0574 | 0.637 | 0.527 |
| 30 mg | (Intercept) | 42.8 | 7.09 | 6.04 | < 0.01 | 46.2 | 6.98 | 6.63 | < 0.01 | 53.9 | 6.71 | 8.02 | < 0.01 | 63.7 | 6.76 | 9.42 | < 0.01 | 53 | 8.31 | 6.37 | < 0.01 |
|  | % reduction of IgE | NA | | | | | | | | | | | | 0.0544 | 0.0326 | 1.67 | 0.104 | NA | | | |
|  | % reduction of TARC | -0.0143 | 0.0306 | -0.468 | 0.641 | 0.0487 | 0.0734 | 0.663 | 0.51 | 0.083 | 0.0435 | 1.91 | 0.0624 | 0.014 | 0.0264 | 0.530204 | 0.599 |  |  |  |  |
|  | % reduction of LDH | 0.604 | 0.296 | 2.03 | 0.0465* | NA | | | | | | | | | | | | 0.703 | 0.407 | 1.72 | 0.0983 |
|  | % reduction of TEC | NA | | | | 0.202 | 0.0905 | 2.23 | 0.0302* | 0.0184 | 0.0872 | 0.211 | 0.833 | 0.06 | 0.07 | 0.86 | 0.395 | 0.0183 | 0.0642 | 0.285 | 0.778 |
| * Statistically significant at *p* < 0.05, ** at *p* < 0.01.  EASI, eczema area and severity index; PP-NRS, peak pruritus numerical rating scale; IgE, immunoglobulin E; TARC, thymus and activation-regulated chemokine; LDH, lactate dehydrogenase; TEC, total eosinophil count; NA, not applicable. | | | | | | | | | | | | | | | | | | | | | |
